# Supplementary material for: TMEM135 regulates primary ciliogenesis through modulation of intracellular cholesterol distribution
Source: EMBO Rep. 2020 Mar 11;21(5):e48901. doi: 10.15252/embr.201948901 (PMC7202201; doi:10.15252/embr.201948901)
Supplement: Supplementary file 1 — Appendix [file EMBR-21-e48901-s001.pdf]

Appendix

Yunash Maharjan[1], Joon No Lee[1], SeongAe Kwak[2], Raghbendra Kumar Dutta[1], Channy Park[1], Seong-Kyu Choe[2], and Raekil Park[1#]

TMEM135 regulates primary ciliogenesis through modulation of intracellular cholesterol distribution

Table of content

1. Appendix Figure S1: TMEM15 localizes to peroxisomes.

2. Appendix Figure S2: TMEM135 depletion is not associated with impaired peroxisomal matrix protein import or accumulation of VLCFA.

3. Appendix Figure S3: TMEM135 siRNA efficiently depletes TMEM135 fluorescent signal from peroxisomes.

4.Appendix Figure S4. Endogenous Rab8 localizes to primary cilium in a small cell population.

5. Appendix Figure S5: TMEM135 depletion mediated impaired Rab8 activation is not associated with Rabin8 dysfunction.

6. Appendix Figure S6: TMEM135 depletion increases peroxisome-Rab8 overlap.

7. Appendix Figure S7: TMEM135 depletion does not affect IFT88 trafficking to centrioles.

8. Appendix Table S1: List of siRNA sequences used in this study.

9. Appendix Table S2: List of antibodies used in this study.

10. Appendix Table S3: List of primer sequence used for RT-qPCR.

11. References

Appendix Figure S1

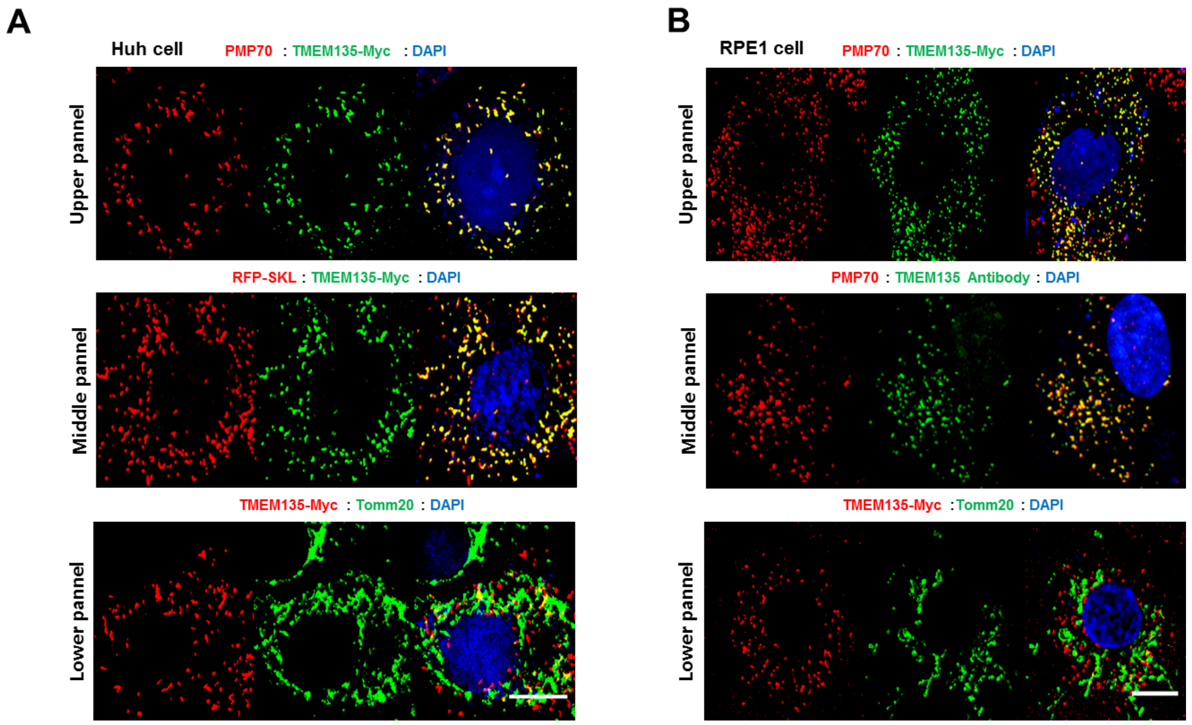

Appendix Figure S1. TMEM135 localizes to peroxisomes.

(A) Huh7 cells were transfected with TMEM135-myc plasmid (Upper and lower panel), followed by immunostaining with antibodies against PMP70 (red) and Myc (green) in the upper panel, Myc (red) and Tomm20 (green) in lower panel. Scale bar, 10 µm. Celle were co-transfected with TMEM135-Myc and RFP-SKL plasmid (middle panel).

(B) RPE1 cells were transfected with TMEM135-Myc plasmids (Upper panel and lower panel) and immunostained with antibodies against PMP70 (red) and Myc (green) in Upper panel and Myc (red) and Tomm20 (green) in lower panel. Scale bar, 10 µm. Endogenous TMEM135 and PMP70 was stained by TMEM135 antibody (green) and PMP70 antibody (red) middle panel. Scale bar, 10 µm.

Appendix Figure S2

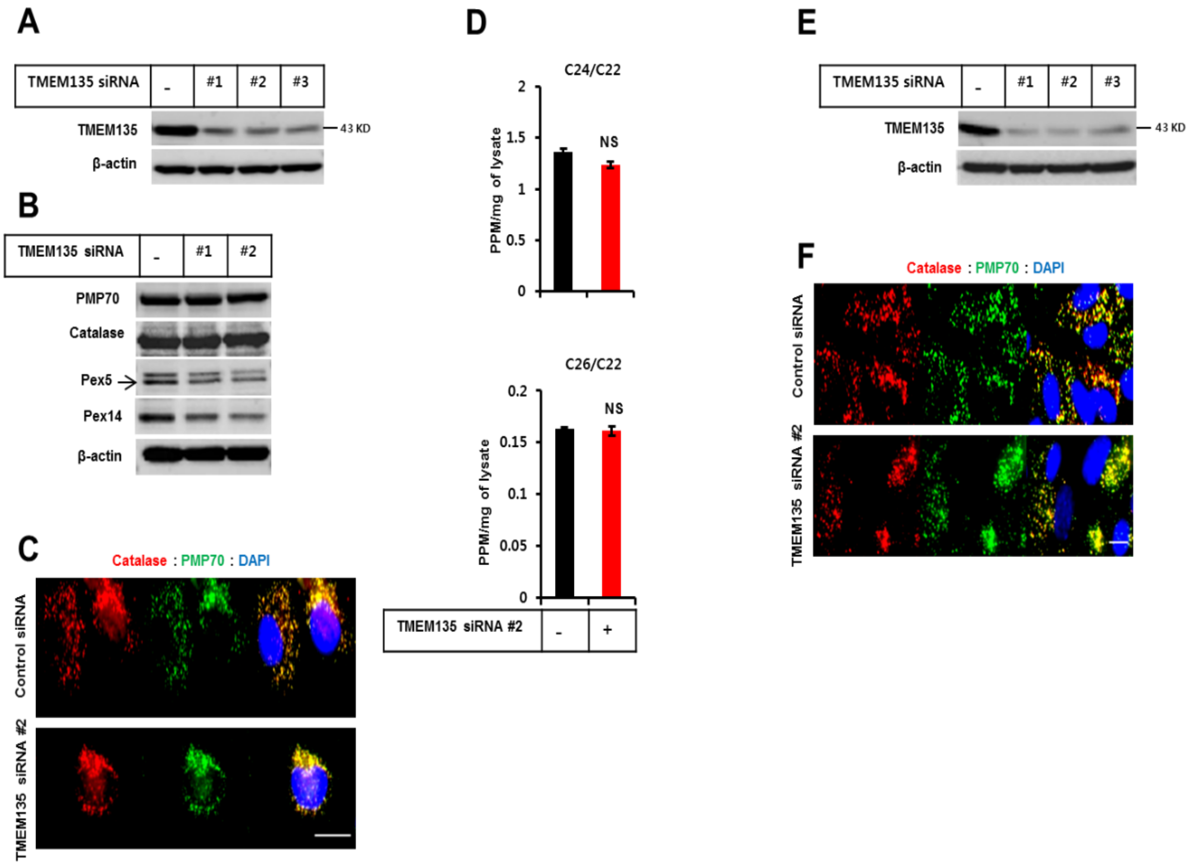

Appendix Figure S2. TMEM135 depletion is not associated with impaired peroxisomeal matrix protein import or accumulation of VLCFA.

(A) Huh7 cells were transfected with siRNAs as indicated followed by Western blot analysis for TMEM135 knock down efficiency by using TMEM135 antibody.

(B) Huh cells were transfected with siRNAs as indicated followed by Western blot analysis of different peroxisome protein (PMP70, catalase, Pex5, Pex14).

(C) Huh cells were transfected with siRNAs as indicated and immunostained with antibodies against catalase (red) and PMP70 (green). Scale bar, 10 µm.

(D) Huh7 cells were transfected with siRNAs as indicated followed by GC-MS analysis of VLCFA. Data represent mean ± SD (n =3 experiments). NS, not significant. Student’s T-test.

(E) RPE1 cells were transfected with siRNAs as indicated followed by Western blot analysis for TMEM135 antibody.

(F) RPE1 cells were transfected with siRNAs as indicated and immunostained with antibodies against catalase (red) and PMP70 (green). Scale bar, 10 μm.

Appendix Figure S3

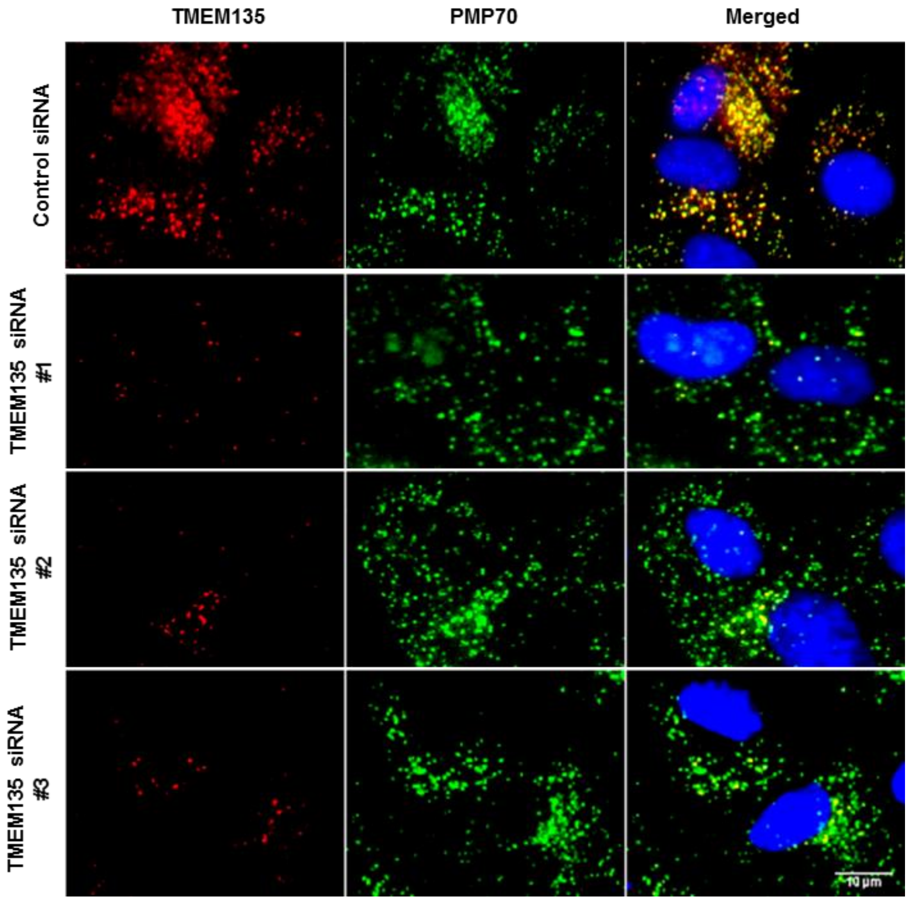

Appendix Figure S3: TMEM135 siRNA efficiently reduces TMEM135 fluorescent signal from peroxisomes. Representative image showing that all three siRNA efficiently reduces TMEM135 protein from peroxisomes stained with PMP70 in RPE1 cell. Scale bar, 10 µm.

Appendix Figure S4

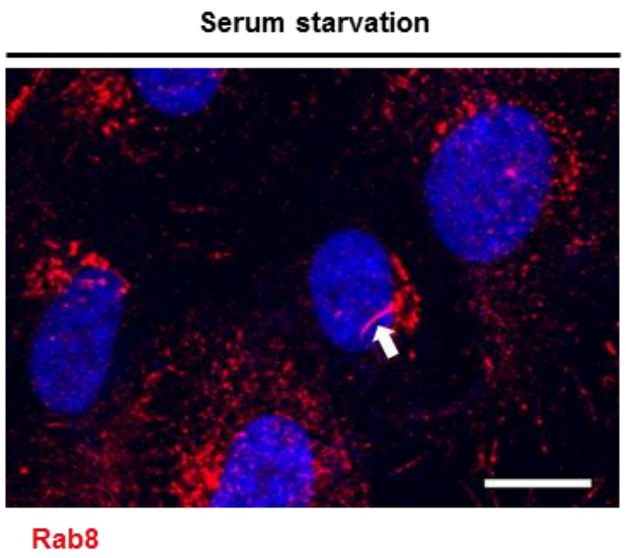

Appendix Figure S4. Few cells were detected with ciliary endogenous ciliary Rab8.

RPE1 cells were serum-starved for 24 h and immunostained with Rab8 antibody. Representative image showing ciliary Rab8. Scale bar, 10 μm. Arrow indicate the endogenous Rab8 localizes in few cell populations during serum-starvation.

Appendix Figure S5

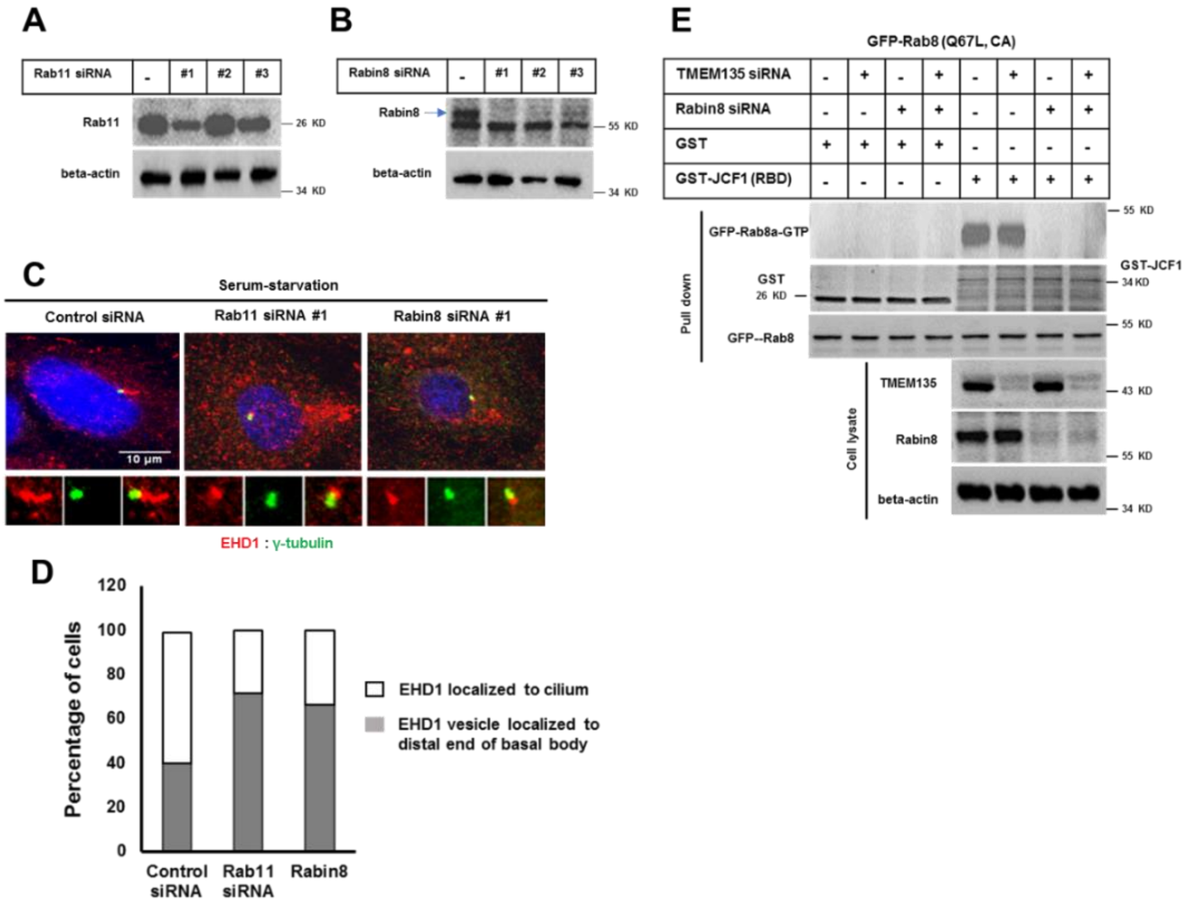

Appendix Figure S5. TMEM135 depletion mediated impaired Rab8 activation is not associated with Rabin8 dysfunction.

(A, B) RPE1 cells were transfected with siRNAs as indicated followed by Western blot analysis for Rab11 and Rabin8 knockdown efficiency respectively.

(C) RPE1 cells were transfected with siRNAs as indicated followed methanol fixation and immunostaining with EHD1 (red) and γ-tubulin (green) antibody. Scale bar, 10 µm.

(D) Quantitative data of **(C)** showing percentage of cells with EHD1 localization in the cilium or basal body. Data represents average (n=2 experiments), 100 cells were scored per condition per experiment.

(E) Cells were transfected as shown and the cell lysates were incubated with purified GST-JCF1 (RBD) fusion protein. The amount of GTP-Rab8 bound to GFT-JCF1(RBD) was analyzed by Western blot with Rab8 antibody.

Appendix Figure S6

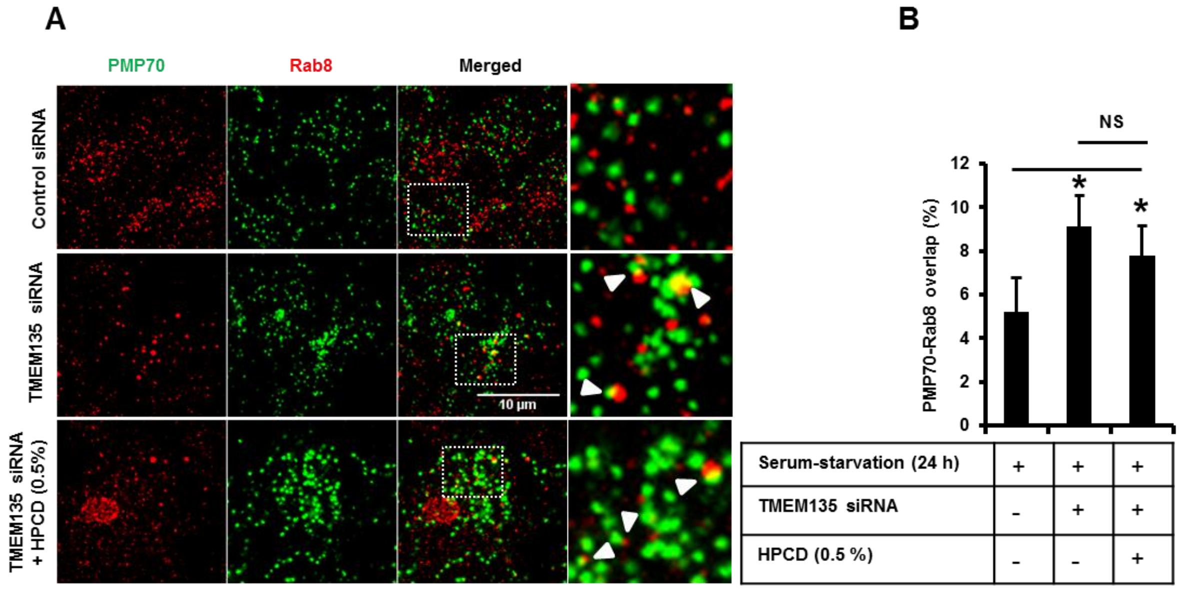

Appendix Figure S6. TMEM135 depletion increases peroxisome-Rab8 overlap.

(A) RPE1 cells were transfected with siRNAs as indicated for 48 h, incubated with/without HPβCD in serum starved media for additional 18 h, and immunostained with antibodies against Rab8 (red) and PMP70 (green). Scale bar, 10 µm.

(B) Quantification of peroxisome-Rab8 overlap in **(A)** Data represent mean ± SD (n = 3 experiments). 50 cells were scored per condition per experiment). *P < 0.05, Student's t-test.

Appendix Figure S7

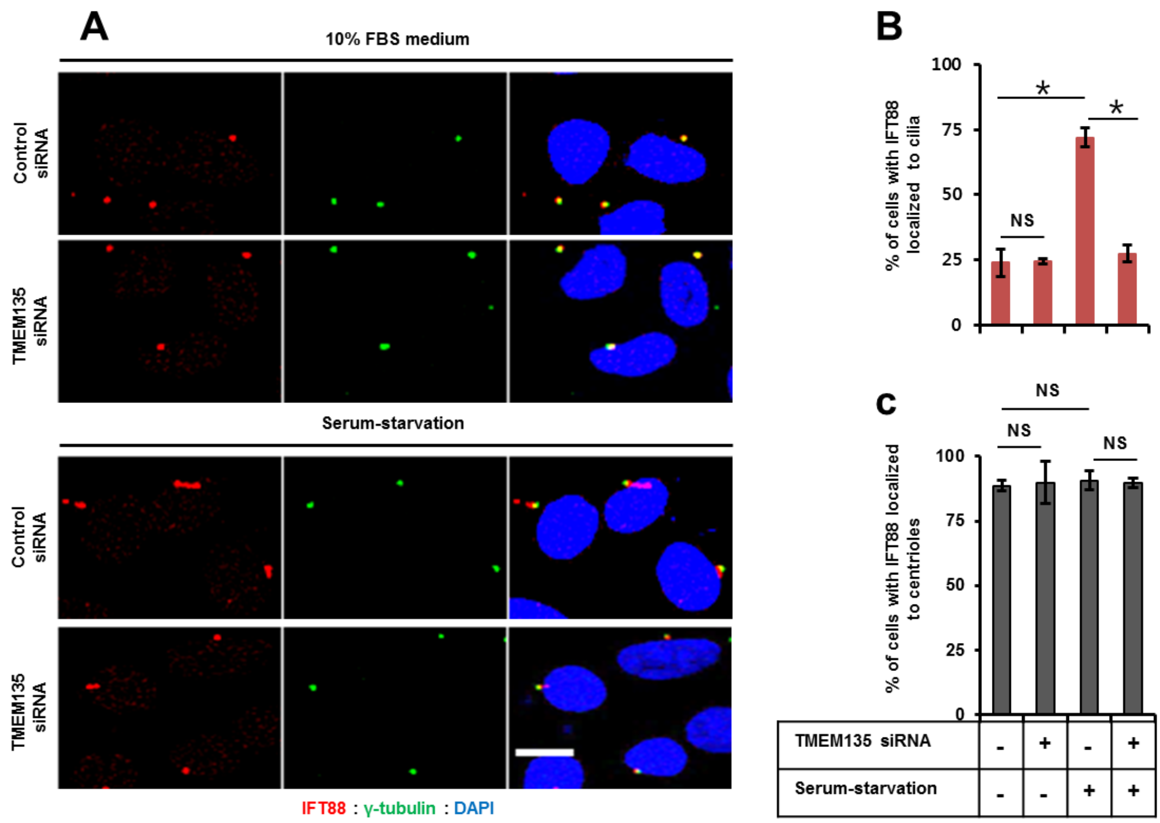

Appendix Figure S7. TMEM135 depletion does not affect IFT88 trafficking to centrioles.

(A) RPE1 cells were transfected with siRNAs as indicated and immunostained with antibodies against IFT88 (red) and γ-tubulin (green). Scale bar, 10 µm.

(B) Quantification of percentage of cells with IFT88 localized to cilium in **(A)** Data represent mean ± SD (n = 3 experiments). 100 cells were scored per condition per experiment *P < 0.05, Student's t-test.

(C) Quantification of percentage of cells with IFT88 localized to centrioles in **(A)** Data represent mean ± SD (n = 3 experiments). 100 cells were scored per condition per experiment. NS, not significant, Student's t-test

Appendix Table S1: List of siRNA sequences used in this study

siRNA	Sense strand sequence	Reference
TMEM135 (1)	5’-GCUCAAAUUAUGCAGUAGA-3’	Bioneer
TMEM135 (2)	5’-AGUUCAGGACAGCUGUGAU-3’	Bioneer
TMEM135 (3)	5’-CUGCAUUUAGGCAUCUGUU-3’	Bioneer
IFT20	5’-GUAAAGUAGAAGCAGAACA-3’	Bioneer
Rab8a	5’-GUAGUGCCAUGAAGCGAUU-3’	Bioneer
Rab8b	5’-CUGUCAUGUUCACUCUGAA-3’	Bioneer
Rab11a (1)	5’-CGAGCUAUAACAUCAGCAU-3’	Bioneer
Rabin8 (1)	5’-GUCAUCAGGACCUCAGUGU-3’	Bioneer

Appendix Table S2: List of antibodies used in this study

Antibody	Application	Dilution	Source	Reference
c-Myc	IF	1:1000	Mouse	sc-40, Santa Cruz Biotech
c-Myc	IF	1:1000	Rabbit	A190-205A, Bethyl
PMP70	IF and WB	1:500	Rabbit	ab3421, Abcam
PMP70	IF	1:500	Mouse	SAB4200181, Sigma
Tomm20	IF	1:500	Mouse	sc-17764, Santa Cruz Biotech
SREBP2	WB	1:1000	Rabbit	Lee JN, et al. (2004)
UBXD8	WB	1:2000	Rabbit	Lee JN, et al. (2010)
CREB	WB	1:2000	Rabbit	#9192, Cell Signaling
CP110	IF	1:500	Rabbit	12780-I-AP, Proteintech
IFT20	IF, and WB	1:500	Rabbit	13615-1-AP, Proteintech
IFT88	IF	1:500	Rabbit	13967-1-AP, Proteintech
Rab8a	IF and WB	1:500 and 1:2000	Rabbit	ab188574, Abcam
ARL13B	IF	1:500	Rabbit	17711-1-AP, Proteintech
Acetelated-tubbulin	IF	1:1000	Mouse	T7451, Sigma
γ-tubulin	IF	1:500	Mouse	T5326, Sigma
GM130	WB	1:1000	Mouse	610822, BD Transduction Laboratories
Rab11	IF	1:200 and 1:2000	Mouse	610656, BD Transduction Laboratoreis
GFP (B-2) antibody	WB	1:1000	Mouse	SC-9996, Santa Cruz Biotech
Rabin8	IF	1:500	Rabbit	12321-I-AP, Proteintech
TCTN1	IF	1:500	Rabbit	15004-I-AP, Proteintech
TMEM135	IF and WB	1:500 and 1:1000	Rabbit	SAB3500832, Sigma
TMEM135	IF and WB	1:500 and 1:1000	Rabbit	Ab222237, Abcam
LAMP1	IF and WB	1:500 and 1:1000	Mouse	sc-2011, Santa Cruz Biotech
Catalase	IF and WB	1:500 and 1:2000	Rabbit	Ab4877, Abcam
Pex5	WB	1:2000	Rabbit	GTX109798
Pex14	WB	1:1000	Rabbit	A303-0858, Bethyl
EEA1	WB	1:1000	Rabbit	C45B10 , Cell signalling
Na^+/K^+ ATPase	WB	1:2000	Rabbit	Sc-21712, Santa Cruz Biotech
EHD1	IF	1:250	Rabbit	HPA067747, ATLAS ANTIBODIES

Appendix Table S3: List of primer sequence used for RT-QPCR

Gene	Primer sequence	Reference
TMEM135	F: 5’-CCAGCTTGGAGCTTTTCTTG-3’	Bioneer
	R: 5’AGTTCATCATCTAAGTTTCTGATCCA-3’	
HMGCoA synthase	F: 5’-GACTTGTGCATTCAAACATAGCAA-3’	Bioneer
	R: 5’-GCTGTAGCAGGGAGTCTTGGTACT-3’	
HMGCoAreductase	F: 5’-CAAGGAGCATGCAAAGATAATCC-3’	Bioneer
	R: 5’-GCCATTACGGTCCCACAC-3’	
LDLR	F: 5’-GGCTGCGTTAATGTGACACTCT-3’	Bioneer
	R: 5’-CTCTAGCCATGTTGCAGACCTTGT-3’	
Insig1	F: 5’-CCCAGATTTCCTCTATATTCGTTCTT-3’	Bioneer
	R: 5’-CACCCATAGCTAACTGTCGTCCTA-3’	
Rab8a	F: 5’-GCCTACTACAGGGGTGCAAT-3’	Bioneer
	R: 5’-GGATGTTGTCGAAGGACTTC-3’	
Rab8b	F: 5’-ACGAGATATAATGACAAAAC-3’	Bioneer
	R: 5’-GATCGGTTTTCTGTTATTT-3’	

References

Lee, J.N., and Ye, J. (2004). Proteolytic activation of sterol regulatory element-binding protein induced by cellular stress through depletion of Insig-1. *J Biol Chem*. **279**, 45257-65.

Lee, J. N., Kim, H., Yao, H., Chen, Y., Weng, K., Ye, J. (2010). Identification of Ubxd8 protein as a sensor for unsaturated fatty acids and regulator of triglyceride synthesis. *Proc Natl Acad Sci USA*. **107**, 21424-9.
