# Supplementary material for: TMEM135 regulates primary ciliogenesis through modulation of intracellular cholesterol distribution
Source: EMBO Rep. 2020 Mar 11;21(5):e48901. doi: 10.15252/embr.201948901 (PMC7202201; doi:10.15252/embr.201948901)
Supplement: Supplementary file 2 — Expanded View Figures PDF [file EMBR-21-e48901-s002.pdf]

Expanded View Figures

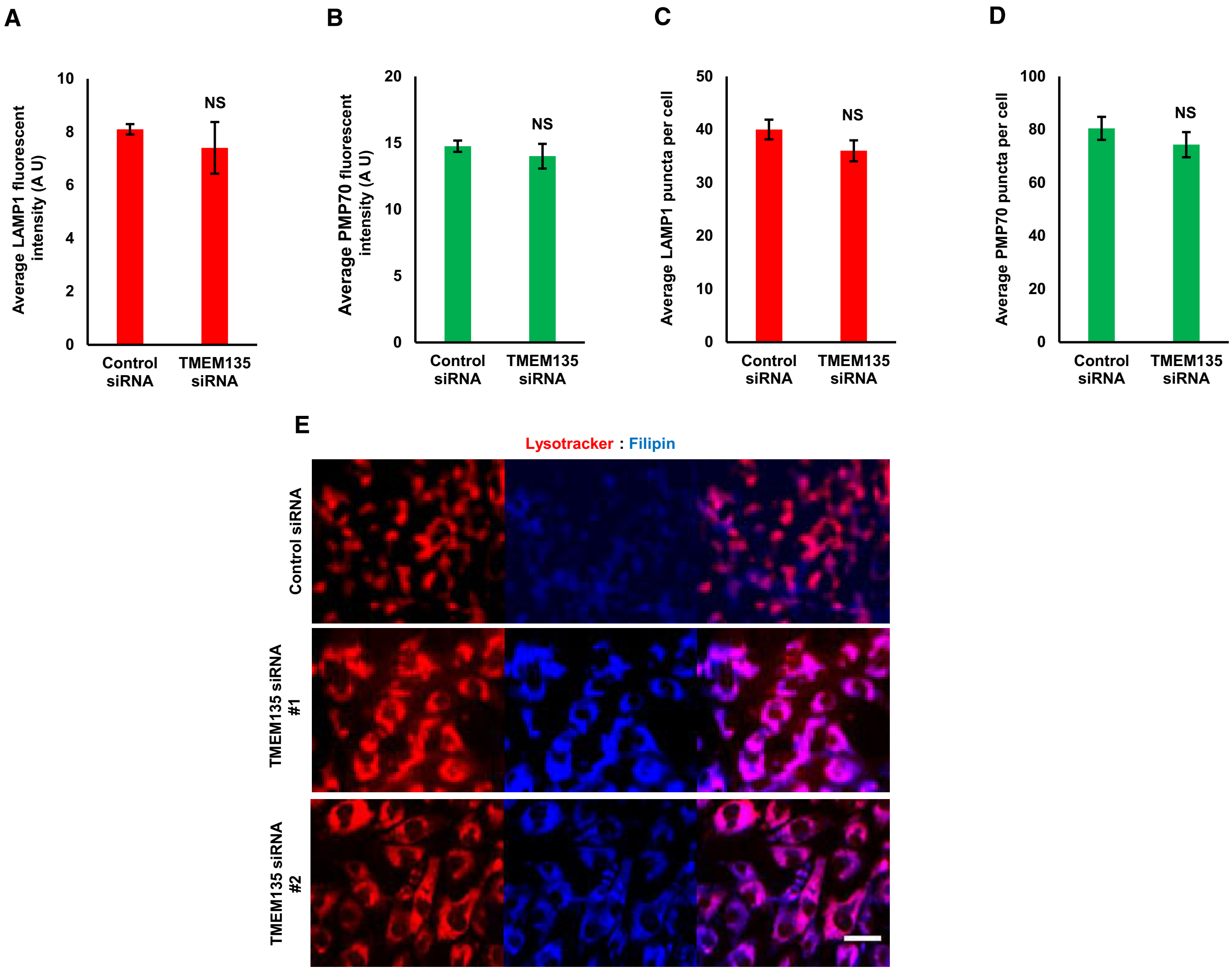

Figure EV1. TMEM135 depletion results in lysosomal cholesterol accumulation without affecting peroxisome and lysosome abundance.

A–D RPE1 cells were transfected with either scramble or TMEM135 siRNAs and immunostained for LAMP1 as a lysosome marker (red) and PMP70 as a peroxisome marker (green). (A, B) Fluorescent intensity of lysosome and peroxisome, respectively, within an area of cell was separately measured by ImageJ software. A. U means arbitrary unit. Data represent mean ± SD (n = 3 experiments), and 50 cells were scored per condition per experiment. NS, not significant; $P > 0.05$, Student's t-test. (C, D) Lysosome and peroxisome puncta were determined within an area of cell. Data represent mean ± SD (n = 3 experiments), and 50 cells were scored per condition per experiment. NS, not significant; $P > 0.05$, Student's t-test.

E RPE1 cells were transfected with siRNAs, followed by sequential staining with lysotracker (red) and filipin (blue). Scale bar, 20 µm.

© 2020 The Authors

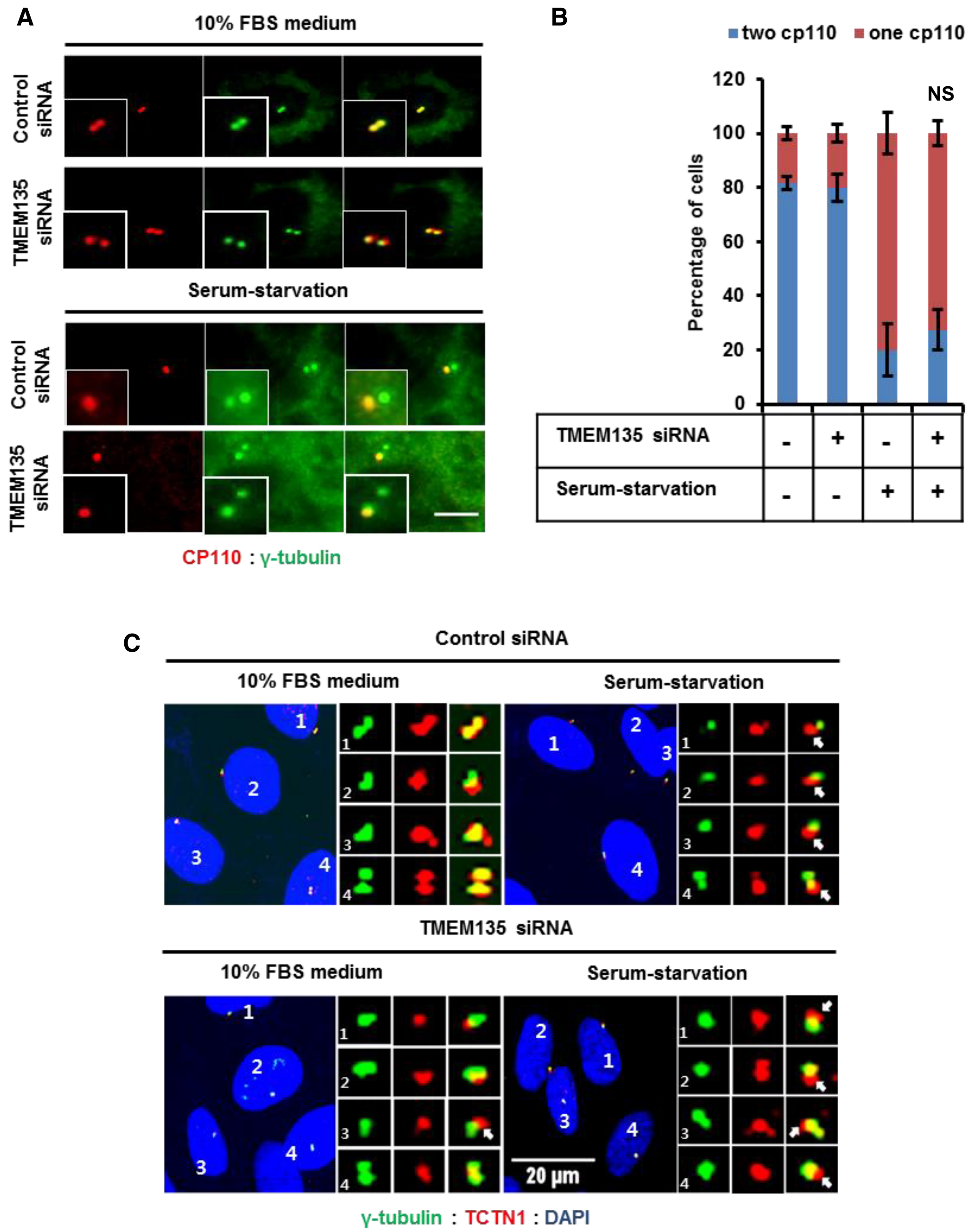

Figure EV2. Basal body uncapping, and transition zone formation is unaffected by TMEM135 depletion.

A RPE1 cells were transfected with siRNAs as indicated and immunostained with antibodies against CP110 (red) and γ-tubulin (green). Scale bar, 10 μm.

B Quantification of percentage of cells with one or two CP110. Data represent mean ± SD (n = 3 experiments), and 200 cells were scored per condition per experiment. NS, not significant; $P > 0.05$, Student's t-test.

C Cells were transfected with siRNAs as indicated and immunostained with antibodies against TCTN1 (red) and γ-tubulin (green). Scale bar, 20 μm.

© 2020 The Authors

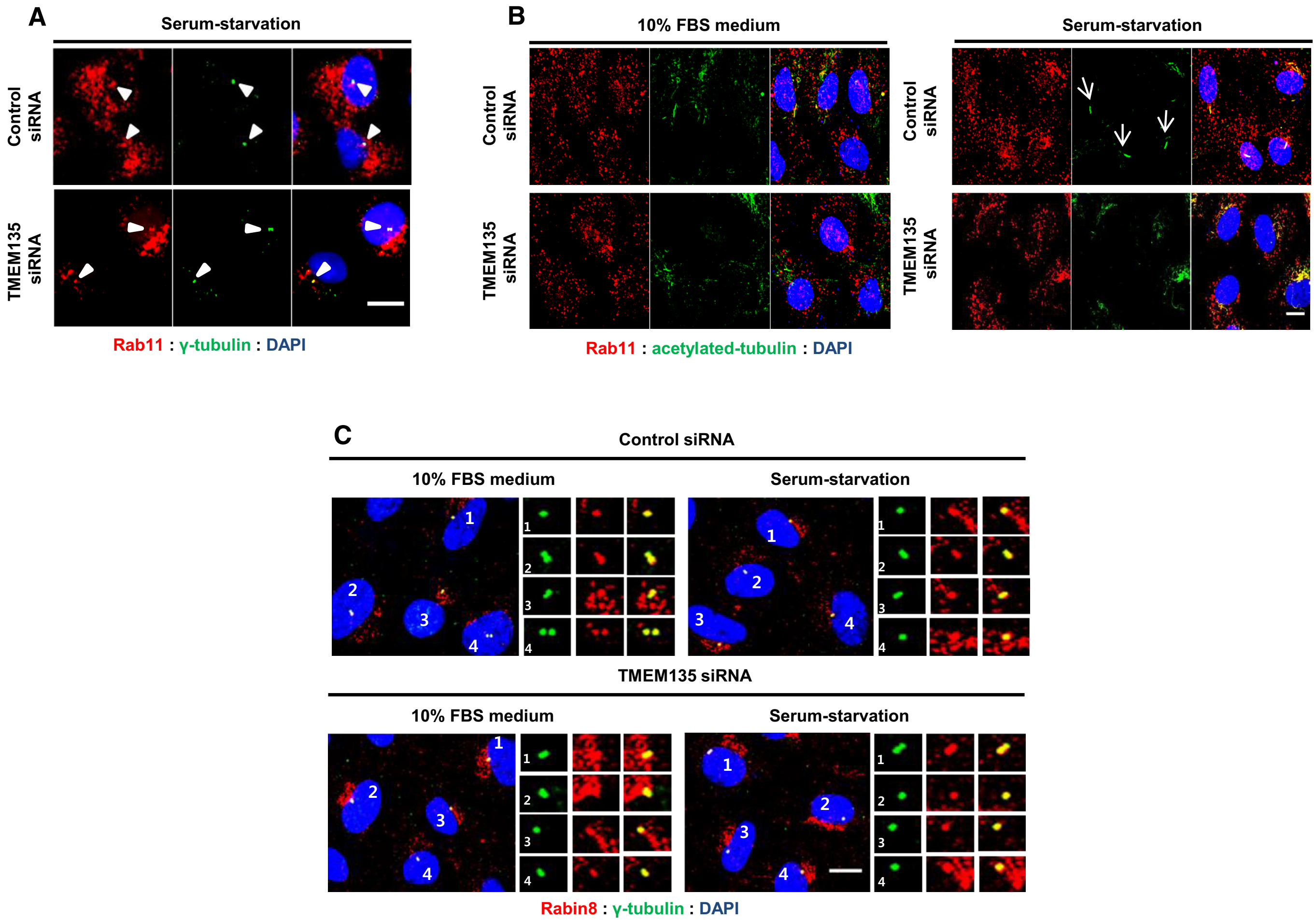

Figure EV3. Impaired ciliogenesis observed in TMEM135-depleted cells is not associated with Rab11 and Rabin8 localization to centrioles.

A RPE1 cells were transfected with siRNAs as indicated and immunostained with antibodies against Rab11 (red) and γ-tubulin (green). Scale bar, 10 μm. Arrowheads showing Rab11 localized to centrioles.

B Cells were transfected with siRNAs as indicated and immunostained with antibodies against Rab11 (red) and acetylated tubulin (green). Scale bar, 10 μm. Arrows showing primary cilium in the serum-starved control cells.

C RPE1 cells were transfected by siRNAs as indicated and immunostained with antibodies against Rabin8 (red) and γ-tubulin (green). Scale bar, 10 μm.

© 2020 The Authors

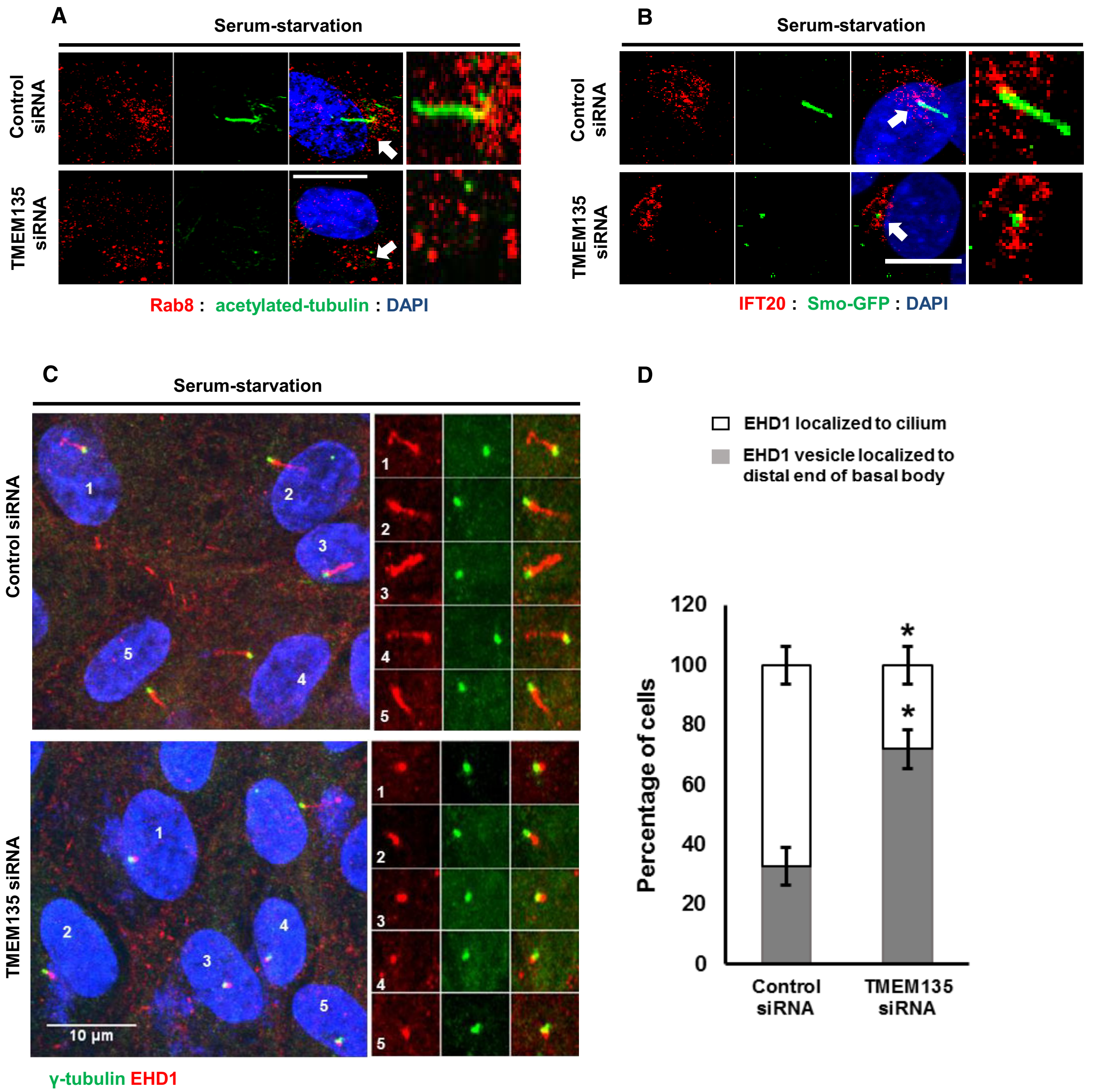

Figure EV4. TMEM135 depletion does not affect ciliary vesicle formation.

A RPE1 cells were transfected with siRNAs as indicated and immunostained with antibodies against Rab8 (red) and acetylated tubulin (green). Scale bar, 10 µm. Arrow showing Rab8 accumulation near the base of the primary cilium in control cells.

B SMO-GFP RPE1 cells were transfected with siRNAs as indicated and immunostained with antibodies against IFT20. Representative confocal images of IFT20 (red) and SMO (green) are shown. Scale bar, 10 µm. Arrow showing the centriolar IFT20 localized at the base of the primary cilium or ciliary vesicle in control and TMEM135-depleted cells, respectively.

C Cells were transfected with siRNAs as indicated and immunostained with antibodies against EHD1 (red) and γ-tubulin (green). Scale bar, 10 µm.

D Quantification of the percentage of cells with EHD1 localized in cilium or at distal end of the basal body (ciliary vesicle). Data represent mean ± SD (n = 3 experiments), and 200 cells were scored per condition per experiment, *$P < 0.05$, Student's t-test.

 © 2020 The Authors

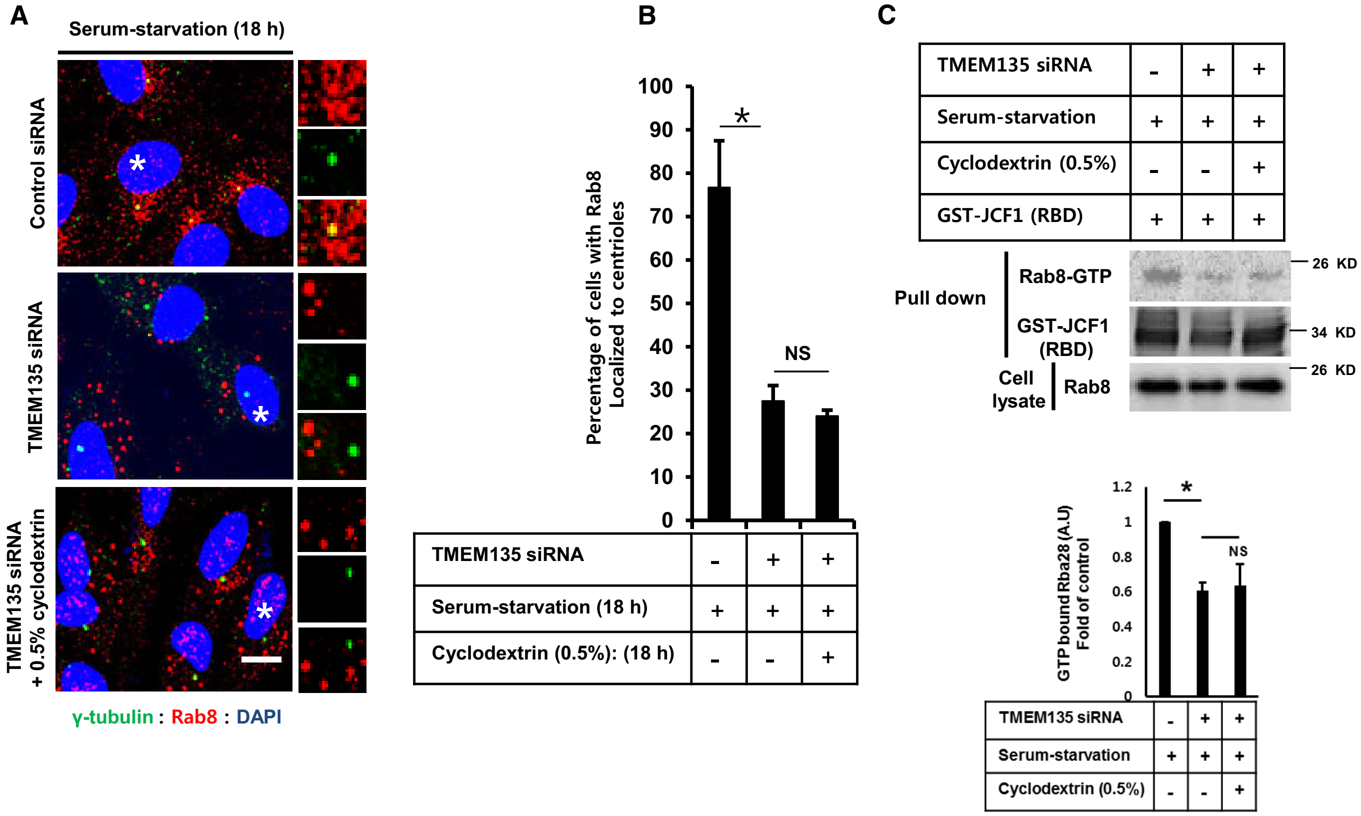

Figure EV5. Removal of cholesterol from lysosomal compartment does not recover impaired Rab8 trafficking and its activation in TMEM135-depleted cells.

A RPE1 cells were transfected with siRNAs as indicated and immunostained with antibodies against Rab8 (red) and γ-tubulin (green). Scale bar, 10 μm. Representative magnified images are shown from cells labeled with white asterisks.

B Quantification of the percentage of cells with Rab8 localized at the centrioles. Data represent mean ± SD (n = 3 experiments), and 200 cells were scored per condition per experiment; *P < 0.05, Student's t-test.

C (Upper panel) Cells were transfected as indicated, and cell lysate was incubated with purified GST-JCF1 (RBD) fusion protein. The amount of GTP-Rab8 bound to GFT-JCF1(RBD) was analyzed by Western blotting with Rab8 antibody. (Lower panel) The intensity of bands was quantified by ImageJ software. The amount of GTP-Rab8 was normalized to the control level. The bar graph represents mean ± SD (n = 3 experiments). *P < 0.05, Student's t-test.

Source data are available online for this figure.

© 2020 The Authors
