# Supplementary figures and images for: TMEM135 regulates primary ciliogenesis through modulation of intracellular cholesterol distribution
Source: EMBO Rep. 2020 Mar 11;21(5):e48901. doi: 10.15252/embr.201948901 (PMC7202201; doi:10.15252/embr.201948901)

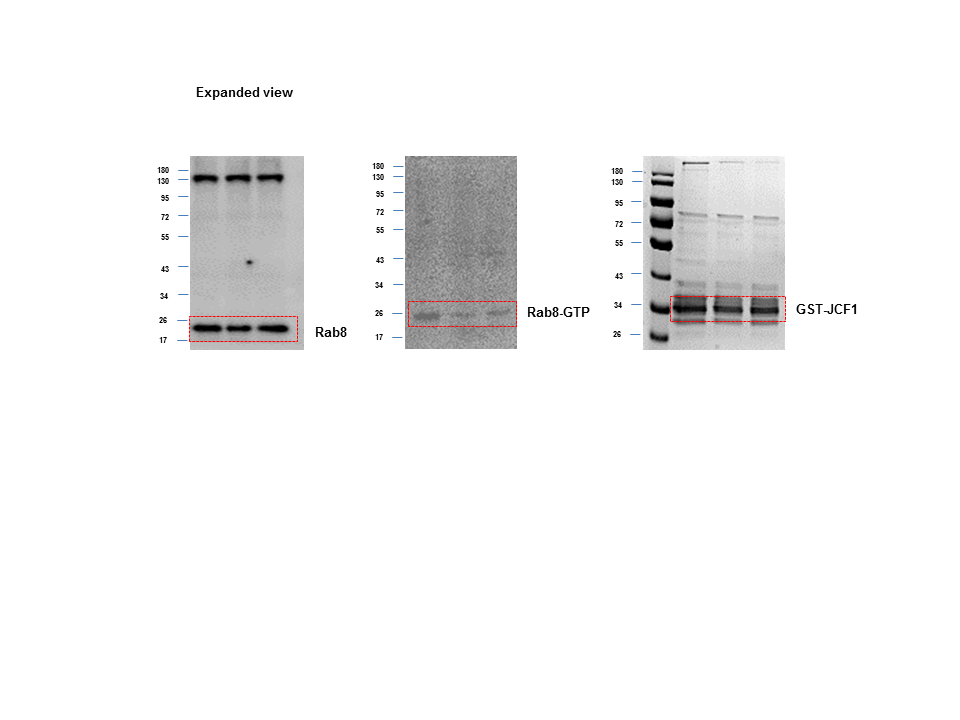

Supplement: Supplementary file 3 — Source Data for Expanded View and Appendix [file EMBR-21-e48901-s008.zip › Source Data Fig EV5.tiff]

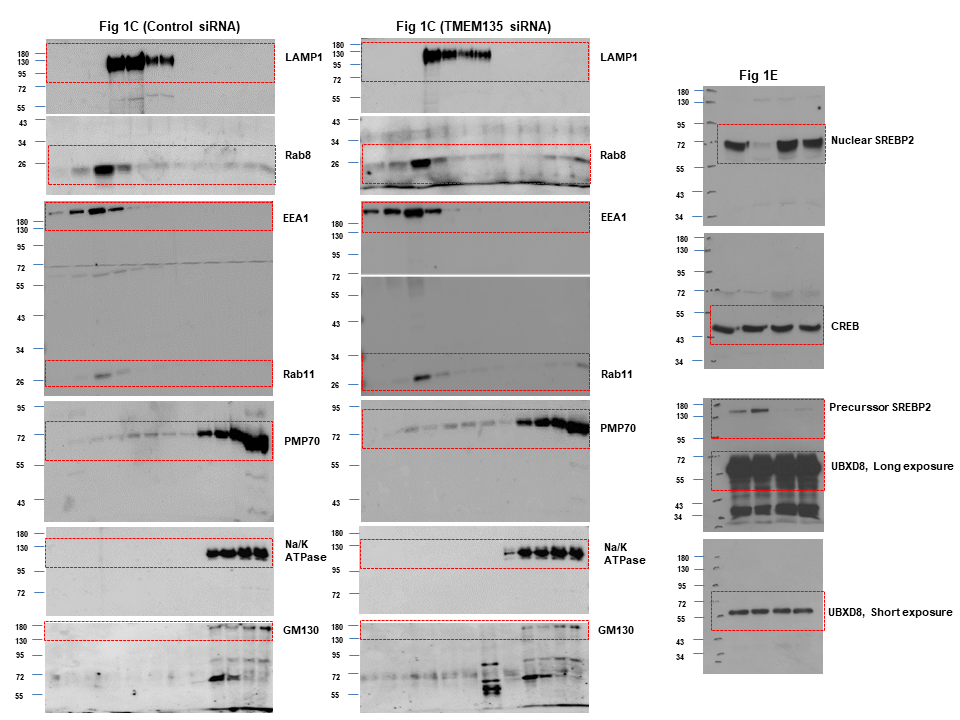

Supplement: Supplementary file 5 — Source Data for Figure 1 [file EMBR-21-e48901-s003.tif]

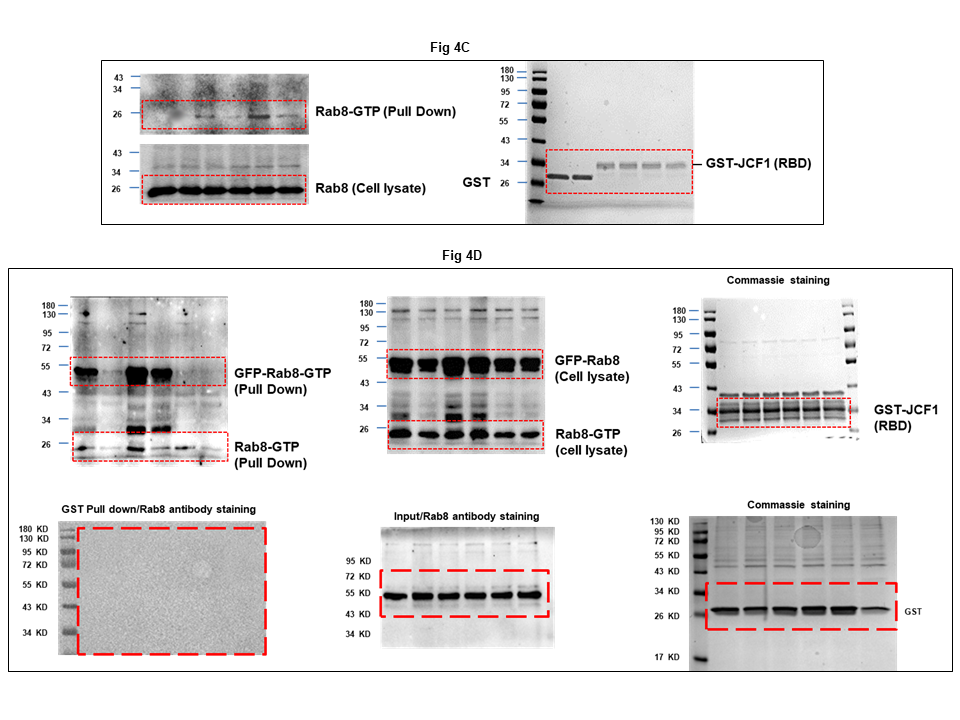

Supplement: Supplementary file 6 — Source Data for Figure 4 [file EMBR-21-e48901-s004.TIF]

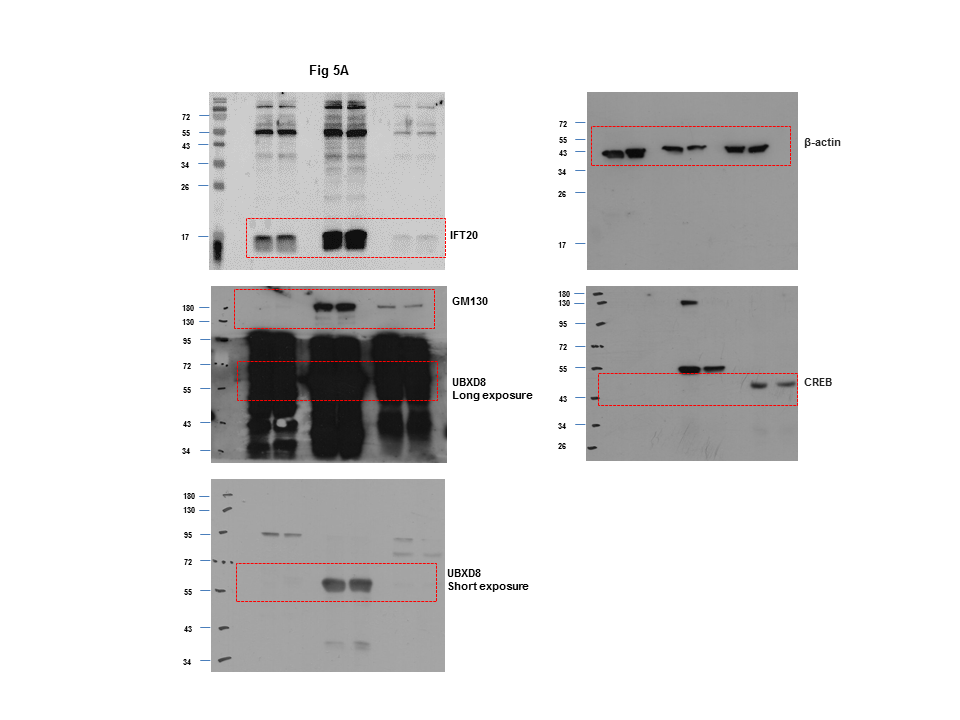

Supplement: Supplementary file 7 — Source Data for Figure 5 [file EMBR-21-e48901-s005.TIF]

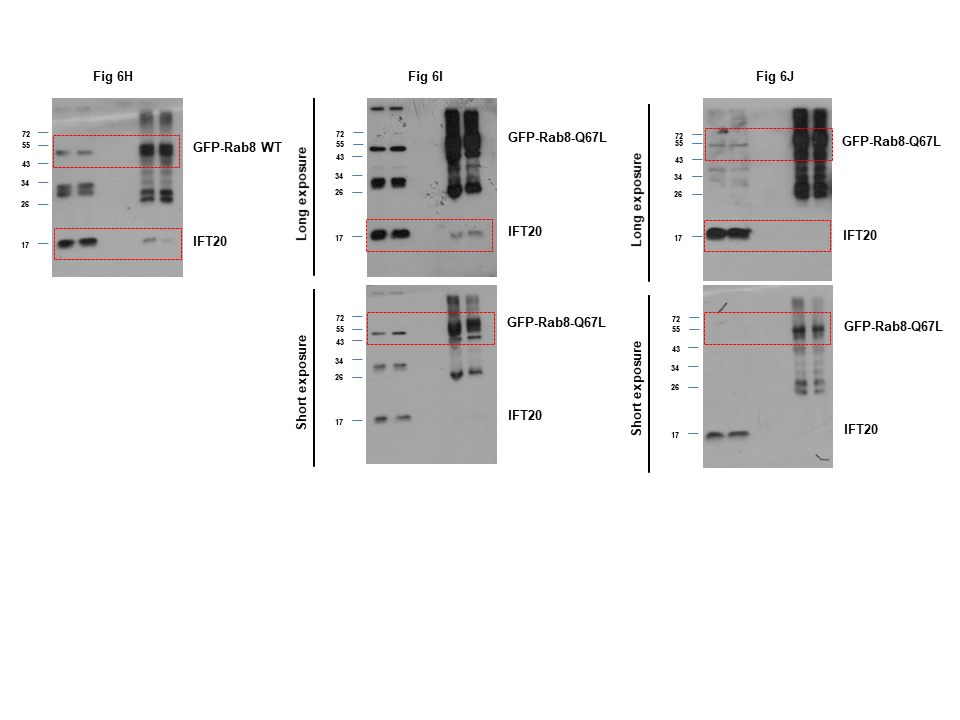

Supplement: Supplementary file 8 — Source Data for Figure 6 [file EMBR-21-e48901-s006.TIF]

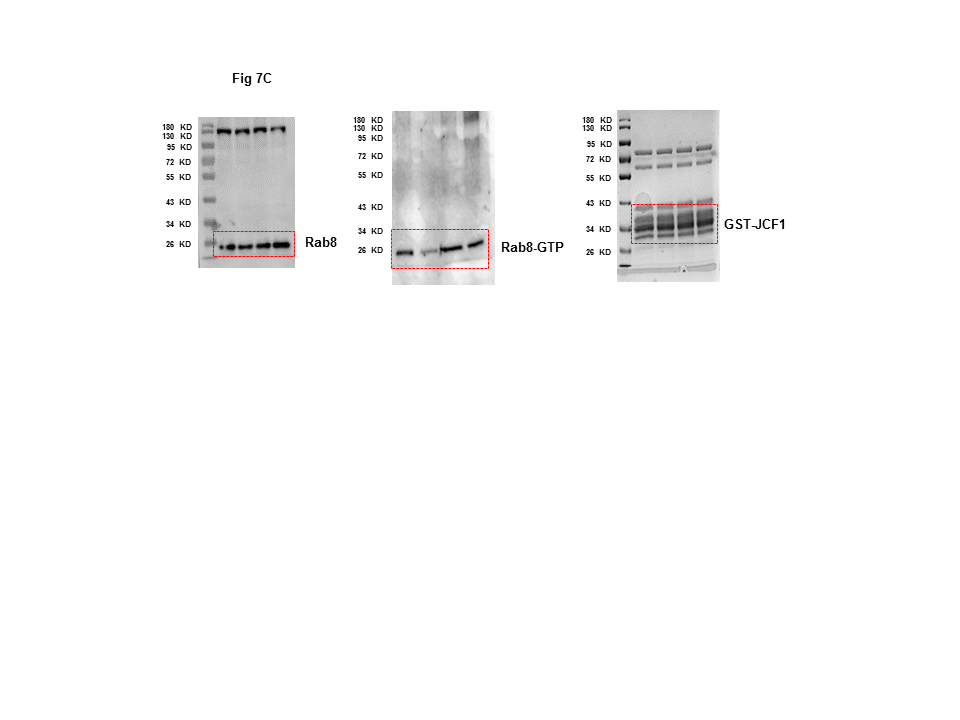

Supplement: Supplementary file 9 — Source Data for Figure 7 [file EMBR-21-e48901-s007.TIF]
